# A New Method of Image Classification Based on Domain Adaptation

**DOI:** 10.3390/s22041315

**Published:** 2022-02-09

**Authors:** Fangwen Zhao, Weifeng Liu, Chenglin Wen

**Affiliations:** 1 School of Electrical and Control Engineering, Shaanxi University of Science and Technology, Xi’an 710021, China; 200611017@sust.edu.cn (F.Z.); liuwf@sust.edu.cn (W.L.); 2 School of Automation, Guangdong University of Petrochemical Technology, Maoming 525000, China

**Keywords:** domain adaptation, unsupervised learning, maximum mean discrepancy

## Abstract

Deep neural networks can learn powerful representations from massive amounts of labeled data; however, their performance is unsatisfactory in the case of large samples and small labels. Transfer learning can bridge between a source domain with rich sample data and a target domain with only a few or zero labeled samples and, thus, complete the transfer of knowledge by aligning the distribution between domains through methods, such as domain adaptation. Previous domain adaptation methods mostly align the features in the feature space of all categories on a global scale. Recently, the method of locally aligning the sub-categories by introducing label information achieved better results. Based on this, we present a deep fuzzy domain adaptation (DFDA) that assigns different weights to samples of the same category in the source and target domains, which enhances the domain adaptive capabilities. Our experiments demonstrate that DFDA can achieve remarkable results on standard domain adaptation datasets.

## 1. Introduction

In recent years, deep learning has achieved great success in computer vision [1] and natural language processing [2] tasks. Unfortunately, satisfactory performance gains come only when large amounts of labeled data are available for supervised training. In practice, it is often time-consuming and expensive to collect enough labeled data or even impossible in some cases, which limits the specific applications of deep neural networks. For a target domain with large amounts of unlabeled data, one natural idea is to transfer the neural network from a domain with richly labeled data to a domain with a shortage of labeled samples. For example, in the fields of medical image diagnosis [3,4] and fault diagnosis [5] where data is scarce, transfer learning is a powerful tool to solve such problems.

Traditional machine-learning algorithms assume that the training data and test data obey the same distribution; however, due to dataset bias, the data distribution on different domains often differs. The generalization ability of the algorithm will be weakened if the statistical distribution of samples is changed. The main idea of domain adaptation [6] is to use a large number of labeled samples from existing domains to facilitate the learning of new tasks by reducing the dataset bias on the target domain.

There are three common approaches to domain adaptation [6,7]: feature-based domain adaptation, instance-based domain adaptation, and classifier-based domain adaptation. The feature-based approach is a typical strategy for domain adaptation [8,9,10,11] and aims to learn a common feature representation by minimizing the distribution discrepancy between domains. The instance weighting method is another simple yet effective domain adaptive approach [12,13,14].

Some instances are selected from the source domain so that the probability distribution of the obtained sample subset is similar to the target sample and are then trained using traditional machine-learning methods. Apart from this, the classifier-based adaptation makes the source domain model adapt to the target by regularizing the difference between the source domain and target domain model parameters [15,16,17]. Since the label of the target domain is required, this usually limits its scope of use.

Previous feature-based domain adaptation methods have mainly used explicit distances to align feature distributions between source and target domains [18,19,20] or minimized the distribution difference between domains by adversarial learning [21,22,23]. These methods align the global source and target domain distribution without considering the category information of the domain samples, which causes some discriminable local information and structures to be confused. Recently, researchers are increasingly investigating subdomain adaptation [24,25,26], which performs local alignment of the source and target domains in the feature space by introducing label information to the neural network. These subdomain-based alignment methods have gained a considerable degree of performance improvement due to capturing the fine-grained information of each category.

**Figure 1:**
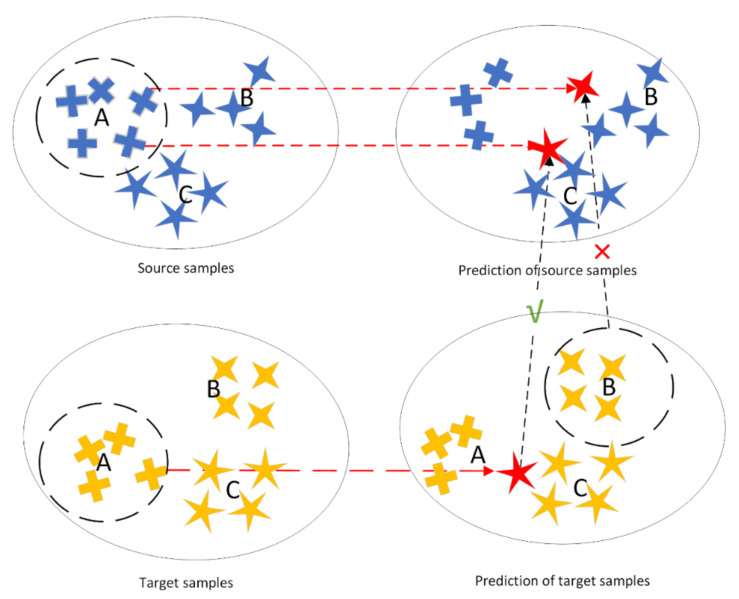
Matching errors may occur when subcategory alignment is performed directly.

Although these aforementioned methods achieved good performance, there is no sample selection process, i.e., alignment of all samples in the same category, which may affect the final results. A straightforward example is shown in Figure 1. During the training period, samples from the source domain category A may be misclassified into the categories B or C after being predicted by the network. If the target domain is aligned with the same category on the source domain, the following incongruities will occur.

For samples with the true label A in the source domain that are misclassified as B or C, it may happen to be aligned with a sample in the target domain that is also misclassified as C, but is more likely to be mismatched with several samples with the true label B. This confusion degrades the ability of domain adaptation. For more accurate alignment, we propose a fuzzy maximum mean discrepancy (FMMD), which considers the predicted information of instances in the source domain to alleviate this problem. Based on this, we design a new network architecture Deep Fuzzy Domain Adaption (DFDA) to apply FMMD, and DFDA can be easily optimized by the standard gradient descent method. The experimental results show that our method outperforms state-of-the-art metric-based approaches on benchmark datasets.

## 2. Related Work

Before discussing the FMMD, in this section, we introduce three related aspects of work: feature-based domain adaptation, subdomain adaptation, and maximum mean discrepancy (MMD).

### 2.1. Feature-Based Domain Adaptation

Research [27] has shown that simple transfer learning methods, such as pretrain-finetune can reduce training time but only slightly improve the result. Feature-based domain adaption embeds adaptive modules into neural networks to reduce the distribution differences between domains and significantly improve the performance of neural networks on the target domain. There are mainly two methods: adversarial learning and statistical moment matching. The domain adaptation method based on adversarial learning brings the idea of GAN (Generative adversarial networks) [28] to the problem of domain adaptation.

The discriminator learns to distinguish between the source domain and target domain features, and the generator learns feature representations with domain invariance to confuse the discriminator. Previous works [24,25,29] have achieved good results. The method based on statistical moment matching measures the difference in distance between domains in terms of the mean or higher order moments and minimizes the difference as much as possible.

MMD reduces the mean between domains in the Reproducing Kernel Hilbert Space (RKHS), CORAL reduces the difference between the covariance matrices the two domains, and CMD (Central moment discrepancy) [30] aligns the higher-order central moments of two domain distributions to perform domain adaptation. Many improved algorithms are subsequently proposed on this basis. Most state-of-the-art methods are domain adversarial net-based adaptation methods. However, domain adversarial networks are often difficult to converge during training, and our method based on the MMD metric also achieves remarkable results.

### 2.2. Subdomain Adaptation

Some recent approaches have improved the performance of domain adaptation by introducing category information into the network. CDAN (Conditional adversarial domain adaptation) [24] conditions the adversarial adaptation model based on the discriminative information in the classifier predictions. MADA (Multi-adversarial domain adaptation) [22] uses a category discriminator for each category, capturing the multimodal structure and fine-grained alignment of the data distribution between domains.

A joint adaptation network (JAN) [31] combines the joint probability distributions of features and labels, aligning both the marginal and conditional distributions of the two domains. A DTN (deep transfer network) [32] uses the output of the discriminator to calculate the conditional MMD and aligns the conditional probability distribution by minimizing the conditional MMD. Deep subdomain adaptation network (DSAN) [26] reduces the MMD distance between samples of the same category on the source and target domains based on the label.

Inspired by the concept of the instance-based transfer method and the different above-mentioned methods, we assign different weights to samples of the same category between domains by adding the predicted value of the samples and selecting some suitable samples for fine alignment. These methods perform domain feature alignment based on the hard pseudo-labeling of samples by the classifier, which will undoubtedly lead to improper alignment due to the presence of noise in these labels.

### 2.3. Maximum Mean Discrepancy

MMD calculates the mean distance of the distribution in the RKHS as the distribution discrepancy between the two domains, and its effectiveness has been proven [33]. There is considerable research around MMD, and the first method to apply MMD to transfer learning was Transfer Component Analysis (TCA) [34]. Deep Domain Confusion (DDC) [35] first adds the MMD to the loss of the deep network feature layer, Deep Adaptation Network (DAN) [36] replaces the MMD with MK-MMD and adapts it in multiple network layers.

Weight MMD (WMMD) [37] alleviates the effect of category imbalance on domain adaptation through class prior distributions. A joint adaptation network (JAN) [31] uses MK-MMD to simultaneously align the marginal distribution and conditional distribution of the two domains. Dynamic Distribution Adaptation (DDA) [38] quantitatively calculates the variability of marginal distribution and conditional distribution between domains based on JAN, and the performance is greatly improved. MMD has become the most popular distance in transfer learning, and DFDA is also based on the MMD approach.

## 3. Methodology

In the unsupervised domain adaptive scenario, we are given a source domain $D_{s}={\left\{x_{i}^{s},y_{i}^{s}\right\}}_{i=1}^{n_{s}}$ with $n_{s}$ labeled samples whose labels $y_{i}^{s}\in Y_{S}$, and target domain $D_{t}={\left\{x_{j}^{t}\right\}}_{j=1}^{n_{t}}$ has $n_{t}$ unlabeled samples. Suppose the source and target domains have the same feature space, i.e., $X_{S}=X_{t}$, and the same category space $Y_{S}=Y_{t}$. $D_{s}$ and $D_{t}$ are sampled from different data distributions P and Q, respectively, and P≠Q. The goal of domain adaptation is how to use the source domain data to build a learner to predict the label of the target domain.

Deep neural networks can learn more transfer representations than traditional machine learning handcrafted features. Several popular deep transfer methods have emerged by adding adaptation layers to neural networks to align the distribution of features between domains [39,40]. These methods reduce the distribution discrepancy between domains by minimizing the distance between the source and target domain features after domain-invariant representations have been extracted by the neural network. However, these methods mainly learn a global domain shift without considering category information to align with subcategories.

DSAN introduces the category information of the source domain and the predicted value information of the target domain into the network and aligns each category separately, which achieves remarkable results on both object recognition tasks and digit classification tasks. However, its assumption that every sample of the same class belongs to class *c* with the same probability $\omega^{c}$, as shown in Figure 1, is unreasonable in some cases. We borrow the idea of an instance-based method to increase the feature weight that is beneficial to the target classification task, decrease the weights of features that are unhelpful to the target classification, and give different weights to each feature to distinguish the importance of the sample, which achieves better performance in the benchmark dataset.

### 3.1. Fuzzy Maximum Mean Discrepancy

Formally, the MMD between distributions P and Q is defined as

(1) MMDHk,P,Q ≔ EfHk≤1sup     X~P∅xis−EY~Q∅xjt

where $H_{k}$ is the reproducing kernel Hilbert space (RKHS) endowed with characteristic kernel *k* (·,·). E[·] denotes the mean of the embedded samples, and $\emptyset \left(\cdot \right)$ denotes some feature map to map the original samples to RKHS. To calculate this difference, an unbiased estimate of MMD is obtained by calculating the squared distance between empirical kernel mean embedding on the sample X and Y instead of sample expectation.

(2) $$\hat{\mathrm{MMD}}\left(H_{k},P,Q\right)=\|\frac{1}{n_{s}}\underset{x_{i}^{s}\in D_{s}}{\sum }\emptyset \left(x_{i}^{s}\right)-\frac{1}{n_{t}}\underset{x_{j}^{t}\in D_{t}}{\sum }\emptyset \left(x_{j}^{t}\right){\|}_{H}^{2}$$

DSAN introduces label information to the network and proposes the Local Maximum Mean Discrepancy (LMMD) to align the distributions of the relevant subdomains within the same category in the source and target domains.

(3) $$\hat{\mathrm{LMMD}}\left(H_{k},P,Q\right)=\frac{1}{C}\overset{C}{\underset{c=1}{\sum }}\|\underset{x_{i}^{s}\in D_{s}}{\sum }\omega_{i}^{sc}\emptyset \left(x_{i}^{s}\right)-\underset{x_{j}^{t}\in D_{t}}{\sum }\omega_{j}^{tc}\emptyset \left(x_{j}^{t}\right){\|}_{H}^{2}$$

where $\omega_{i}^{sc}$ and $\omega_{j}^{tc}$ denote the weight of $x_{i}^{s}$ and $x_{j}^{t}$ belonging to class *c*, respectively. Assuming that the weight of each sample in the same category is equal:

(4) $$\omega_{i}^{c}=\frac{1}{\sum_{\left(y_{i}\in C)\right.}y_{i}}\;$$

However, there are gaps in quality between samples, and the alignment of outliers can easily become confusing. In particular, when a category is too sparse in a batch, the sample weight is prone to unreasonable assignments. To address this issue, we propose the FMMD as follows:

(5) $$\hat{\mathrm{FMMD}}\left(H_{k},P,Q\right)=\frac{1}{C}\overset{C}{\underset{c=1}{\sum }}\|\underset{x_{i}^{s}\in D_{s}}{\sum }\omega_{i}^{sc}{\hat{y}}_{i}\emptyset \left(x_{i}^{s}\right)-\underset{x_{j}^{t}\in D_{t}}{\sum }\omega_{j}^{tc}\emptyset \left(x_{j}^{t}\right){\|}_{H}^{2}$$

where ${\hat{y}}_{i}$ is the output of the source domain samples after the neural network, which represents the confidence that $x_{i}$ belongs to class *c*. It is used to evaluate the quality of the features here. If the predicted value of the sample is closer to the label value, it means that it is a good sample and increases the weight of the sample. On the contrary, if the predicted value deviates from the true value, the feature weight of this sample will decrease. As in DSAN, the weights on the target domain sample are calculated using the predicted values instead of the true label.

### 3.2. Deep Fuzzy Domain Adaption

**Figure 2:**
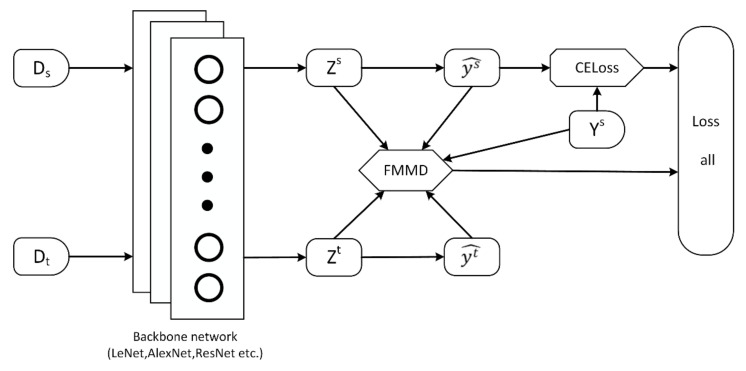
The architecture of DFDA, where CELoss is the cross-entropy loss function.

We propose a new network architecture Deep Fuzzy Domain Adaption (DFDA) in order to embed FMMD into the network. Different from the previous method that only uses the label of the source domain, the predicted information of the source domain samples are also added to the network. As shown in Figure 2, DFDA can be trained end-to-end by the standard stochastic gradient descent method. The loss of the entire network is as follows:

(6) $$\ell_{\mathrm{loss}}=\ell_{C}\left(x^{s},y^{s}\right)+\lambda D\left(z^{s},z^{t},y^{s},\hat{y^{s}},\hat{y^{t}}\right)$$

where $\ell_{C}\left(x^{s},y^{s}\right)$ denotes the loss of source domain data in the neural network. As $\ell_{C}\left(x^{s},y^{s}\right)$ becomes smaller, the accuracy of the source domain is constantly improving. $D\left(z^{s},z^{t},y^{s},\hat{y^{s}},\hat{y^{t}}\right)$ denotes the FMMD metric function; $z^{s}$ and $z^{t}$ denote the feature vectors output by $x^{s}$ and $x^{t}$ via the neural network, respectively; and $\hat{y^{s}}$ and $\hat{y^{t}}$ denote the predicted results of the samples on the source and target domains, respectively. As the feature divergence between the target and source domains becomes smaller, the accuracy of target domain prediction increases with the increase of the source domain sample accuracy.

### 3.3. Theoretical Analysis

We analyzed the effectiveness of DFDA based on the domain adaptation theory [39,41].

**Theorem** **1.** Let H be a hypothesis space of VC dimension d. Given two domains *S* and *T*, then, for any $\delta \in \left(0,1\right)$ with probability at least 1−δ, for every h∈H:

(7) $$\epsilon_{T}\left(h\right)\leq \epsilon_{S}\left(h\right)+\frac{1}{2}d_{H\Delta K}\left(S,T\right)+\lambda \;$$

$\epsilon_{S}\left(h\right)$ and $\epsilon_{T}\left(h\right)$ are the empirical errors in the source and target domains, respectively. where $\epsilon_{S}\left(h\right)$ can be easily minimized by the label information of the source domain sample. $\lambda =\epsilon_{S}\left(h^{*}\right)+\epsilon_{T}\left(h^{*}\right)$ denotes the combined error of the ideal hypothesis, where $h^{*}=\underset{h\in H}{\mathrm{argmin}\;}\epsilon_{S}\left(h\right)+\epsilon_{T}\left(h\right)$ represents an ideal joint hypothesis that achieves the minimum combined errors on both the source and target domains. If combined error is large, there will not be a classifier that performs well in both the source and target domains; therefore, we usually assume that λ is a relatively small and negligible value.

In this case, the second term, $d_{H\Delta K}\left(S,T\right)$, which represents the distribution difference between the source domain and the target domain, is an important component to constrain the error bounds in the target domain. DFDA aligns relatively high-quality samples so that the target domain and the source domain’s similar samples are more accurately matched. Compared with the previous method, $d_{H\Delta K}\left(S,T\right)$ will become smaller. In summary, utilizing the prediction of the source samples in unsupervised domain adaptation is effective.

## 4. Experiment

This section mainly introduces the data sets and experimental environment used in the experiment and describes in detail the analysis. We evaluate our algorithm on three widely used benchmark datasets, including the Office31, Office Home, and large-scale digital recognition datasets, and compare it with several state-of-the-art distance metric-based transfer learning models: DDC, DAN, JAN, Deep CORAL, and DSAN, to assess the effectiveness of our approach.

### 4.1. Setup

Office31 [42] is one of the most widely used datasets for the domain adaptation, and it contains three domains: Amazon (A), Webcam (W), and DSLR (D), with 2817, 498, and 795 samples, respectively, with each including 31 object classes. The samples in Amazon are downloaded from the Amazon website, the samples in Webcam are low-resolution images taken by surveillance equipment, and the samples in DSLR are high-resolution images taken by SLR cameras. We evaluate all methods across all six tasks A→W, W→A, W→D, D→W, A→D, and D→A as DSAN.

Office Home [43] is much larger than the Office31 data set. Each domain has 65 object categories and contains a total of 15,588 color images in office and home scenarios. These images come from four domains: artistic images (A), clip art (C), product images (P), and real-world images (R). Similarly, we use all domain combinations and construct 12 transfer tasks.

Digital recognition dataset contains three widely used benchmarks: MNIST [44], USPS [45] and SVHN [46], MNIST contains 60,000 training images and 10,000 test images, and USPS contains 7291 training images and 2007 test images, all the images in MNIST and USPS are 28 × 28 grayscale images. Here, we follow the settings in DSAN and JDA, and randomly sample 2000 and 1800 images in MNIST and USPS, respectively, to form a new dataset. SVHN contains 32 × 32 color images, but each image may contain multiple digits. We conduct experiments on three transfer tasks MNIST→USPS, USPS→MNIST, and SVHN→MNIST.

### 4.2. Implementation Detail

**Figure 3:**
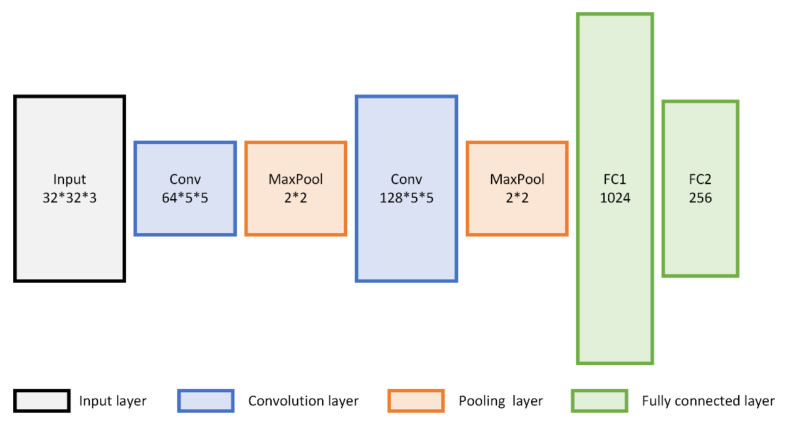
The structure of the modified LeNet model.

For the digital recognition dataset, all the images are resized to 32 × 32 as the input of the network. We use the modified LeNet [42] as the feature extraction network, which mainly contains two convolutional layers with a convolution kernel size of 5 × 5, followed by two fully connected layers fc1 and fc2 with 1024 and 256 units, respectively, and uses the output of fc2 as inputs of FMMD. The modified LeNet model is shown in Figure 3.

For the other two datasets, we follow the settings in DSAN and employ ResNet50 as the feature extraction network, and a bottleneck layer fcb with 256 units is added after the last average pooling layer to reduce the dimensionality. Finally, we use the output of fcb as the FMMD input. We use the pre-trained model on ImageNet to fine-tune all convolutional and pooling layers, and we train the classifier layer via backpropagation.

We follow the settings of DSAN. For each task, we use a mini-batch stochastic gradient descent (SGD) with a momentum of 0.9, and the weight attenuation coefficient is 5 × 10^−4^. The learning rate is adjusted during SGD using the following formula: $\eta_{p}=\eta_{0}/{\left(1+10p\right)}^{0.75}$, where p is the training progress linearly varying from 0 to 1, $\eta_{0}=0.01$, which is optimized to promote convergence and low error on the source domain. Instead of fixing the adaptation factor $\lambda_{p}$, we dynamically adjust it via Equation (8).

(8) $$\lambda_{p}=\frac{2}{1+\exp\left(-10p\right)}-1$$

This progressive strategy can effectively suppress noisy activations at the early stages of training.

For a fair comparison of the above methods, the same network architecture is used on the same dataset. We implement these with publicly available code (https://github.com/jindongwang/transferlearning/ accessed on 27 September 2021) and report the average classification accuracy and standard error for three random trials. For all MMD-based approaches, we adopt a Gaussian kernel, and the bandwidth is set to the median pairwise squared distance on the training data.

### 4.3. Results

Table 1, Table 2 and Table 3 show the results of the different methods on the digital recognition dataset, Office-31, and Office Home, respectively. DFDA outperforms the compared methods on most tasks. There is a 1.7% improvement on the digital recognition dataset and an average accuracy improvement of more than 0.8% on the Office-31 dataset. These facts show that our approach is indeed effective and enhances the domain adaptive capability compared to DSAN.

We also observed only about a 0.6% performance improvement in the Office Home dataset. The main reason is that, in the batch size of 64 samples, compared to the digital recognition dataset with only 10 categories, the probability of each class of sample present in the Office Home dataset with 65 categories is much smaller. That is, the former expects about 6.4 occurrences per category, while the latter is less than 1. This means that there are more samples available for quality selection in the digital recognition dataset, and thus the improvement is the largest. In conclusion, our method performed better in larger batch sizes compared with smaller batches.

### 4.4. Parameter Sensitivity Analysis

**Figure 4:**
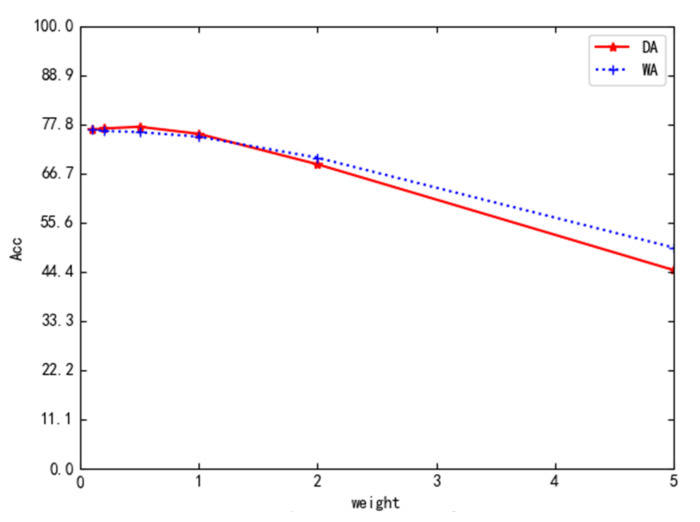
Performance of DFDN on task DA and WA with different $\lambda_{p}$.

Although the weight coefficient $\lambda_{p}$ of the transfer loss is changed in the experiment, we also studied the effects of different $\lambda_{p}$. Figure 3 demonstrates the variation of average accuracy of DFDA on tasks D**→**A and W**→**A for $\lambda_{p}\in \left\{0.1,0.2,0.5,1,2,5\right\}$. As shown in Figure 4, the accuracy improves slightly with increasing $\lambda_{p}$ and then decreases; however, good results can be achieved in the range of less than 1.

**Figure 5:**
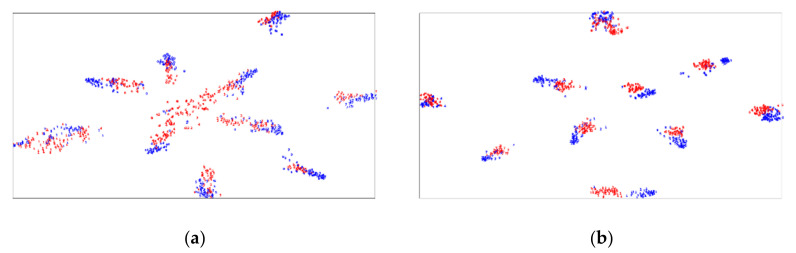
(**a**,**b**) The feature visualization results of DSAN and DFDA for the SVHN→MNIST task, respectively.

To demonstrate the effect of transfer learning, we utilize t-SNE (https://lvdmaaten.github.io/tsne/ accessed on 12 October 2021) to visualize in Figure 5a,b the network activations of task SVHN→MNIST learned by DSAN and DFDA. Blue points are source samples, and red are target samples. Figure 5a shows the representations learned by DSAN, and it can be seen that although some categories can be well matched, there are still some that are confused. In contrast, the same categories on the source and target domains are properly aligned in Figure 5b. It is clear from the figure that our results are better than DSAN. We can find that the source and target domains are not well aligned using DSAN and some points are hard to classify. The main reason is that DSAN did not conduct quality assessment of the samples and align all samples, which caused confusion.

### 4.5. Discussion on the Advantage of DFDA

To give an overview of the results, we compared DFDA with several other MMD-based metrics in terms of the execution time and computational cost. The performance test was performed on a computer with a NVIDIA RTX3090 GPU, and the results are shown in Table 4.

Our approach does increase some matrix operations over the previous approach; however, these computational costs are negligible compared to the computational effort brought by ResNet50, and considerable performance gains can be obtained. The average increase in computing time per epoch is only 0.3 s, which we believe is worth the cost.

## 5. Conclusions

The previous subdomain adaptive approach aligns all samples within the relevant subdomain without considering the quality differences of individual features. In this paper, we proposed a new method of DFDA to measure the distance between domains, which evaluates the quality of individual features in the source domain by adding predicted information of the source domain to the network. Particularly in the setting of a large batch size, this can select more suitable samples among multiple same-class samples on the source domain to match with the target domain, which effectively improved the performance of the transfer model. Compared with previous methods, our proposed DFDA achieved 1.7% and 0.8% performance improvements on the digital recognition and Office-31 datasets, respectively, and these results support the effectiveness of our proposed method.

## Figures and Tables

**Table 1:** Accuracy (%) on digit recognition tasks for unsupervised domain adaptation.

Method	MNIST→USPS	USPS→MNIST	SVHN→MNIST	Avg
baseline	94.5	76.0	71.2	80.5
DDC	96.0	95.3	77.5	89.6
Deep CORAL	96.0	87.7	72.4	85.4
DSAN	97.2	**97.1**	87.7	94.0
DFDA	**97.5**	**97.1**	**92.5**	**95.7**

**Table 2:** Accuracy (%) on Office-31 for unsupervised domain adaptation (resnet50).

Method	A→W	D→W	W→D	A→D	D→A	W→A	Avg
ResNet50	68.4 ± 0.5	96.7 ± 0.5	99.3 ± 0.1	68.9 ± 0.2	62.5 ± 0.3	60.7 ± 0.3	76.1
DDC	75.8 ± 0.2	95.0 ± 0.2	98.2 ± 0.1	77.5 ± 0.3	67.4 ± 0.4	64.0 ± 0.5	79.7
Deep CORAL	77.7 ± 0.2	97.6 ± 0.2	99.7 ± 0.1	81.1 ± 0.4	64.6 ± 0.4	64.0 ± 0.3	80.8
JAN	85.4 ± 0.3	97.4 ± 0.2	99.8 ± 0.2	84.7 ± 0.3	68.6 ± 0.3	70.0 ± 0.4	84.3
DSAN	**93.6 ± 0.2**	98.3 ± 0.1	**100 ± 0.0**	90.2 ± 0.7	73.5 ± 0.5	74.8 ± 0.4	88.4
DFDA	93.5 ± 0.3	**98.7 ± 0.1**	**100 ± 0.0**	**90.3 ± 0.5**	**75.9 ± 0.4**	**76.8 ± 0.2**	**89.2**

**Table 3:** Accuracy (%) on Office-Home for unsupervised domain adaptation (resnet50).

Method	A→C	A→P	A→R	C→A	C→P	C→R	P→A	P→C	P→R	R→A	R→C	R→P	Avg
ResNet	34.9	50.0	58.0	37.4	41.9	46.2	38.5	31.2	60.4	53.9	41.2	59.9	46.1
DAN	43.6	57	67.9	45.8	56.5	60.4	44.0	43.6	67.7	63.1	51.5	74.3	56.3
DANN	45.6	59.3	70.1	47.0	58.5	60.9	46.1	43.7	68.5	63.2	51.8	76.8	57.6
JAN	45.9	61.2	68.9	50.4	59.7	61.0	45.8	43.4	70.3	63.9	52.4	76.8	58.3
DSAN	54.4	70.8	75.4	60.4	67.8	68.0	**62.6**	55.9	78.5	73.8	60.6	83.1	67.6
DFDA	**54.8**	**71.4**	**75.5**	**61.8**	**69.7**	**68.6**	62.5	**56.6**	**79.0**	**74.2**	**61.2**	**83.7**	**68.2**

**Table 4:** The performance of different methods on the Office-31 dataset. (Batch size = 64).

	ResNet50	DDC	DSAN	DFDA
FLOPs	4.2 × 10^9^	12,845,086	13,635,232	13,639,200
Epoch time (s)	21.9	32.1	32.3	32.5
Accuracy (%)	76.1	79.7	88.4	89.2
